# Mobile Apps to Reduce Tobacco, Alcohol, and Illicit Drug Use: Systematic Review of the First Decade

**DOI:** 10.2196/17156

**Published:** 2020-11-24

**Authors:** Petra Karin Staiger, Renee O'Donnell, Paul Liknaitzky, Rachel Bush, Joanna Milward

**Affiliations:** 1 School of Psychology Deakin University Faculty of Health Geelong Australia; 2 Centre for Drug Use, Addictive and Anti-social behaviour Research (CEDAAR) Deakin University Geelong Australia; 3 Monash Centre of Health Research and Implementation Monash University Clayton Australia; 4 Department of Addictions Institute of Psychiatry, Psychology and Neuroscience Kings College London London United Kingdom

**Keywords:** smartphone app, mobile phone, mobile app, problematic substance use, addiction, systematic review, mHealth, ecological momentary intervention, alcohol, tobacco, smoking, illicit drugs

## Abstract

**Background:**

Mobile apps for problematic substance use have the potential to bypass common barriers to treatment seeking. Ten years following the release of the first app targeting problematic tobacco, alcohol, and illicit drug use, their effectiveness, use, and acceptability remains unclear.

**Objective:**

This study aims to conduct a systematic literature review of trials evaluating mobile app interventions for problematic tobacco, alcohol, and illicit drug use.

**Methods:**

The review was conducted according to recommended guidelines. Relevant databases were searched, and articles were included if the mobile app study was a controlled intervention trial and reported alcohol, tobacco, or illicit drug consumption as outcomes.

**Results:**

A total of 20 studies met eligibility criteria across a range of substances: alcohol (n=11), tobacco (n=6), alcohol and tobacco (n=1), illicit drugs (n=1), and illicit drugs and alcohol (n=1). Samples included the general community, university students, and clinical patients. The analyzed intervention sample sizes ranged from 22 to 14,228, and content was considerably diverse, from simple stand-alone apps delivering self-monitoring or psychoeducation to multicomponent apps with interactive features and audio content, or used as adjuncts alongside face-to-face treatment. Intervention duration ranged from 1 to 35 weeks, with notifications ranging from none to multiple times per day. A total of 6 of the 20 app interventions reported significant reductions in substance use at post or follow-up compared with a comparison condition, with small to moderate effect sizes. Furthermore, two other app interventions reported significant reductions during the intervention but not at post treatment, and a third reported a significant interaction of two app intervention components.

**Conclusions:**

Although most app interventions were associated with reductions in problematic substance use, less than one-third were *significantly* better than the comparison conditions at post treatment. A total of 5 out of the 6 apps that reported intervention effects targeted alcohol (of those, one targeted alcohol and illicit drugs and another alcohol and tobacco) and 1 targeted tobacco. Moreover, 3 out of 6 apps included feedback (eg, personalized) and 2 had high risk of bias, 1 some risk, and 3 low risk. All 6 apps included interventions of 6 weeks or longer. Common study limitations were small sample sizes; risk of bias; lack of relevant details; and, in some cases, poorly balanced comparison conditions. Appropriately powered trials are required to understand which app interventions are most effective, length of engagement required, and subgroups most likely to benefit. In sum, evidence to date for the effectiveness of apps targeting problematic substance use is not compelling, although the heterogeneous comparison conditions and trial designs across studies limit the ability to compare efficacy between apps. We discuss potential approaches that can help ascertain whether the promise of mobile app interventions for problematic substance use can be fulfilled.

## Introduction

The problematic use of substances such as alcohol, tobacco, and illicit drugs is one of the leading causes of morbidity and mortality worldwide [1]. Despite the devastating health and social consequences, a large proportion of individuals who engage in problematic substance use do not seek formal treatment [2-4]. Help-seeking barriers include concern about anonymity, not knowing about or being able to access treatment, and the financial or time burdens of treatment [5-8]. Hence, interventions that can address some of these help-seeking barriers warrant attention to reduce the substantial negative impact of substances at a population level.

Mobile health (mHealth) interventions purport to overcome many of these help-seeking barriers by offering a population-based approach, improving access and affordability [9,10]. mHealth refers to health support delivered on mobile devices, such as cell phones, smartphones, and tablets [11], typically using dedicated apps, but also includes systems such as interactive voice response (IVR) and text messaging (SMS) [12,13]. Apps have rapidly become the most popular software method for delivering health support (ie, mHealth) on mobile devices. As of August 2019, a search of iTunes and Google Play indicated that over 45,000 mHealth apps are currently available. Surprisingly, despite the plethora of apps available to assist people in reducing problematic alcohol and other drug use, only a very small proportion of these apps are evidence based [14]. Evaluations of other health-related apps, for example in the field of mental health, have produced positive [15] or negligible [16] changes in the targeted behavior. Importantly, a number of early trials of apps that focused on problematic substance use have produced promising results, suggesting that apps could play a role in assisting individuals who are dependent on tobacco, alcohol, or illicit drugs to quit or maintain abstinence [17].

It has been approximately 10 years since the emergence of mobile apps designed to help people reduce or recover from problematic alcohol, tobacco, and/or illicit drug use [18]; 6 years since controlled trials have appeared [17]; and 5 years since the first publication of a systematic literature review of 6 smartphone apps for problematic substance use [19]. Of note, all but 1 review has examined the literature on digital interventions more broadly, and all reviews have only included alcohol interventions within their review. For example, Kaner et al [20] found that digital interventions (ie, delivered via a computer, smartphone, or mobile device) for alcohol use showed they significantly lowered alcohol consumption, with an average reduction of up to 3 (United Kingdom) standard drinks per week compared with control participants. Similarly, a meta-analysis showed that internet-delivered alcohol interventions significantly reduced problematic drinking behavior among adults, reducing by 5 standard units of alcohol consumption each week compared with the control group [21]. Berman et al [22] examined the use of mobile interventions (IVR, SMS, and apps) to reduce drinking in university students. A total of 2 of the 7 reviewed studies used apps, and they found that only an IVR intervention resulted in a reduction in the primary outcome. Finally, the most recent related review focused on alcohol use in community participants and similarly included a range of mobile interventions beyond apps [23]. Moreover, 5 of the 19 studies in their review [23] included app-based interventions with mixed findings reported. To date, there has been only 1 app-specific systematic review, which included pilot studies and open trials [19], which evaluated the alcohol app studies across community and alcohol-dependent individuals. The authors found that 2 of the 6 mobile apps reviewed reported reliable positive outcomes, with a further 2 showing promise. The authors highlighted the limited number of studies, small sample sizes, lack of control groups, and limited rigorous designs within the field of mobile app interventions for problematic substance use. Since their review, which was conducted in 2015, there has been a three-fold increase in controlled evaluations of mobile apps designed to reduce substance use or aid recovery from substance dependence. Although some reviews have included apps when examining the effectiveness of alcohol interventions delivered via mobile devices [20,22,23], surprisingly, there has been no further synthesis of the evidence regarding the effectiveness of problematic alcohol use interventions delivered specifically via mobile apps. Moreover, none of the app-specific reviews included smoking and illicit drug use to develop a comprehensive *picture* of the effectiveness of apps across the substance use field. Thus, this paper reports on the current evidence base regarding the effectiveness and feasibility of mobile apps designed to reduce problematic alcohol, tobacco, and illicit drug use. In addition, we report usability, adherence, retention, and engagement data where possible. This information is critical to gain a deeper understanding of user experience and behavior alongside effectiveness data. Thus, it has been approximately 10 years since the first appearance of an app targeting the reduction of substances, and it is timely for us to review the progress of the field.

## Methods

### Aims and Guidelines

A systematic search of the literature was performed to synthesize the findings on effects, retention, and usability from primary studies evaluating mobile app interventions to reduce tobacco, drug, and/or alcohol use. An initial scan of the literature indicated that there was a wide range of mobile delivery methods, considerable variability in intervention content, and control groups; hence*,* we decided a priori to not conduct a traditional meta-analytic review given the potential risk of drawing premature conclusions. The review focused on primary consumption outcomes (ie, quantity and/or frequency of substance consumption) rather than related harm or secondary psychosocial outcomes. The search followed the PRISMA (preferred reporting items for systematic review and meta-analyses) guidelines [24]. Data extraction was guided by the CONSORT-EHEALTH (Consolidated Standards of Reporting Trials-Electronic Health Checklist) [25]. Risk of bias was assessed using the *Risk of Bias* tool developed by the Cochrane Collaboration [26].

### Literature Search and Screening

The literature search utilized the following large databases: MEDLINE (Medical Literature Analysis and Retrieval System Online), PsycInfo, EMBASE (Excerpta Medica dataBASE, via the OVID platform), and ERIC (Education Resources Information Center, via EBSCO). The databases were searched using variations of 3 key terms: Substance AND Intervention AND Smartphone App (refer to Multimedia Appendix 1 for the detailed search strategy).

### Eligibility Criteria

The search was limited to papers published in peer-reviewed journals from January 1, 2007 (the year when the first smartphone was released) to February 1, 2019. Papers were eligible if they adhered to the following criteria: (1) they reported primary empirical data; (2) the primary focus of the intervention was reduction in the use of illicit drugs, and/or alcohol, and/or tobacco; (3) substance consumption outcome data were reported; (4) the intervention was delivered via a mobile device app (not web-based or SMS in isolation); and (5) a controlled trial design was employed using either a randomized or matched control methodology. There were no limitations on the type of control conditions employed. In addition, no language restrictions were imposed.

**Figure 1:**
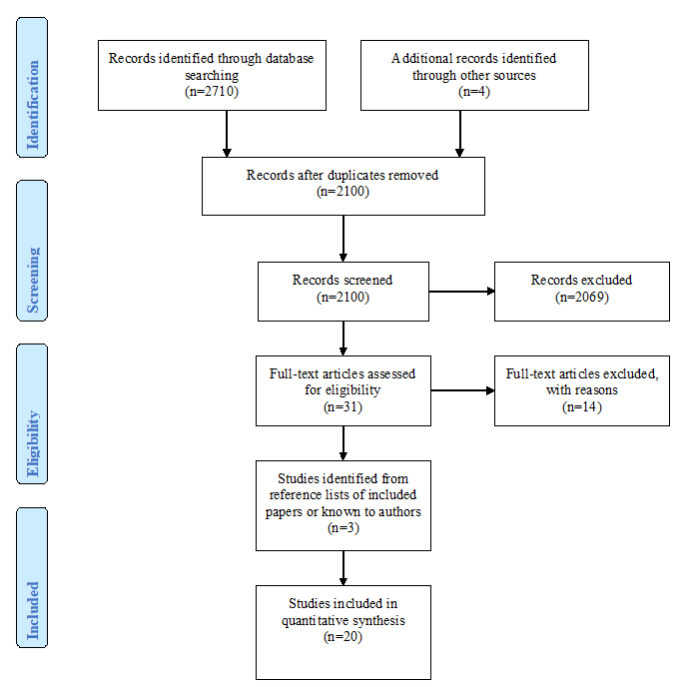
Flow diagram illustrating the selection process for the systematic review of the literature.

The software program Covidence (Veritas Health International) [27] was utilized to ensure independence of screening and accurate calculation of agreements. As shown in Figure 1, the combined search identified a total of 2714 potentially relevant articles (4 papers sourced from examining the reference lists of existing reviews) that reduced to 2100 after duplicates were removed. In keeping with the methodology proposed by Moskowitz [28] and Foxcroft et al [29], an abstract and title search was conducted independently on all papers by 2 people (KG and RO) with a random 20% of these cross-checked (by PS). Any disagreements were discussed by the authors (PS, RO, and PL) and an agreement made, resulting in a total of 31 potentially relevant articles. These 31 papers were read independently and in full by RO and PS. During this process, 14 additional articles were excluded. Agreement regarding exclusions was high (84%), with only 2 disagreements, which were then discussed with PL to reach a final decision. Each of the reference lists of the remaining papers were scanned, and papers known to the authors were included, which identified 3 additional studies, bringing the total number of studies informing this review to 20.

### Data Extraction

The following data were extracted (RB and RO) from the 20 eligible articles: year, authors, study sample, target substance, consumption measure, length of intervention, description of the intervention and control and associated sample sizes, assessment times, summary of statistical evidence, effect sizes where possible (Tables 1 and 2). As indicated earlier, the only outcome data extracted were consumption variables, given that it is the most consistently reported variable, thus enabling comparability between studies. In this respect, means and SDs for all consumption outcomes for each group at baseline, post intervention, and follow-up (if reported) were extracted as well as the relevant statistical data and effect sizes (if reported). Some information was gained via contacting authors, and this is noted in Table 2. Retention and usability data were also extracted to provide an informed discussion regarding the feasibility of delivering interventions for substance use via an app (Table 3). Considerable data are reported in these tables, and hence, only brief summaries are reported in the text.

**Table 1:** Summary of studies.

Author, date [reference]; “app name”	Sample type; target substances	Intervention groups description	Comparison groups description	Intervention duration; assessment time points^a^; retention^b^	Age; gender
*Aharonovich et al, 2017 [30]; “HealthCall”* ^c^	HIV-positive adults, drug and alcohol use during past month; drugs and alcohol	“HealthCall” consists of (a) Self-monitoring questions: primary drug; other drugs; drinking; HIV medication adherence; safe sex practices; wellness, stress. (b) Personalized feedback: graphs with goal attainment and feedback. (c) Option to call counselor. App use: daily notifications for 2 months. Adjunct components: (a) Motivational interviewing: face-to-face session at baseline, and two booster sessions.	Motivational interviewing as described for intervention group. Comparison includes app? No	Duration: 60 days; assessments: baseline, 60 days; retention: 91%	Whole sample: mean 50.96 (SD 7.04) years; 23.40% female
Baskerville et al, 2018 [31]; “Crush the Crave”	Young adults who smoked cigarettes daily and were considering quitting smoking in the next 30 days; tobacco	“Crush the Crave”: (a) graphic performance feedback, (b) evidence-based information on relapse, craving management, (c) notifications on rewards, app use reminders; (d) access to quit lines, and (e) use of nicotine replacement therapy. App use: At will, with prompts to use the app. Adjunct components: “Crush the Craving” Facebook community; app delivered support and inspirational photos tailored to quit plan and stage of quitting; recording smoking so they understand triggers.	Printedself-help guide, “On the Road to Quitting”; (a) health benefits of quitting, (b) rewards of quitting, (c) smoking triggers, (d) coping with withdrawal and cravings, (e) setting a quit date, (f) seeking counseling and use of nicotine replacement therapy, (g) information on social support, (h) telephone support, (i) prevention of weight gain, and (j) relapse Comparison includes app? No	Duration: 6 months; assessments: baseline, 3 months, 6 months; retention: 61%	Intervention: aged range 19-29 years; 44.9% female; comparison: age range 19-29 years; 47.0% female
Boendermaker et al, 2015 [32]; “Alcohol/Avoid”	University students reporting regular drinking; alcohol	“Alcohol/Avoid” (a) cognitive bias modification using alcoholimages; (b) participants swipe alcohol images away, and soft drink images toward themselves. App use: 14 days of access. Adjunct components: none.	Desktop computer version of Alcohol/Avoid training. Comparison includes app? No	Duration: 14 days; assessments: baseline, 28 days; retention: 81%	Whole sample: mean 22.44 (SD 2.58) years; 60.32% female
Bricker et al, 2014 [33]; “SmartQuit”	Adults who smoke at least five cigarettes daily; tobacco	“SmartQuit” consists of Acceptance Commitment Therapy and: (a) motivation and planning to quit, (b) acceptance of urges, and (c) self-compassion for slips. App use: 8-weeks access, no notifications, weekly emails. Adjunct components: none.	Use of app “QuitGuide” from National Cancer Institute. Similar features but no acceptance or self-compassion components: (a) reasons and plans to quit, (b) coping with slips and urges, and (c) staying positive. Comparison includes app? Yes.	Duration: 8 weeks; assessments: baseline, 2 months; retention: 82%	Intervention: mean 41.5 (SD 12.0) years; 53% female; comparison: mean 41.6 (SD 13.9) years; 51% female
Crane et al, 2018 [34]; “Drink Less”	Adults with AUDIT^d^>8, risky drinkers; alcohol	“Drink Less”–enhanced version: (a) goal setting, (b) personalized normative feedback, (c) cognitive bias retraining, (d) self-monitoring and feedback, (e) action planning, and (f) identity change.^e^ App use: daily notifications to report consumption for 4 weeks, optional use of intervention modules. Adjunct components: none.	“Drink Less” minimal version consists of (a) goal setting; (b) alcohol psychoeducation; (c) sham cognitive bias retraining; (d) consumption self-monitoring; (e) information only about action planning; (f) information only on role of identity. Comparison includes app? Yes.^f^	Duration: 4 weeks; assessments: baseline, 4 weeks; retention: 27%	Both conditions: mean 39.2 (SD 10.9) years; 56% female
*Crane et al, 2018 [35]; “Smoke Free”*	Smokers aged over 18 years who had set a quit date (no consumption criteria)	“Smoke Free” full version (a) goal setting and (b) delivery of messages that report on the benefits achieved since cessation attempt, iand “Daily Missions,” which included behavior change techniques to resist cravings. App use: Daily messages delivered for 30 days from quit date. Adjunct components: none.	“Smoke Free” reduced version (a) goal setting; (b) self-monitoring; (c) delivery of messages of the benefits achieved since cessation attempt. Comparison includes app? Yes.	Duration: 12 weeks; assessments: baseline, 12 weeks; retention: 8%	Intervention: mean 28.7 (SD 9.0) years; 49% female; comparison: mean 29.1 (SD 9.4) years; 49% female
Davies et al, 2017 [36]; “Drinks Meter”	Adults aged 18-30 years who self-identified as a current drinker; alcohol	“Drinks Meter” consists of (a) Personalized feedback compared to individuals with similar demographics about alcohol use, calories consumed, and money spent. (b) Assessment (AUDIT) and brief advice and strategies regarding reduction. App use: At will, not stated whether prompts were sent. Adjunct components: none.	Comparison 1: website consists of (a) 20 questions about embarrassing events experienced while drinking and (b) tailored feedback on consequences of drinking. Comparison 2: control website consists of (a) being asked to imagine receiving information about alcohol. Comparison 3: assessment only. Comparison includes app. No.	Duration: 4 weeks; assessments: baseline, 4 weeks; retention: 82%	Whole sample: mean 21.70 (SD 3.28) years; 67.20% female
*Earle et al, 2018 [37]; “CampusGANDR”*	University students (no consumption criteria); alcohol	App intervention 1: “CampusGANDR” (PNF^+^) uses normative feedback and peer judgement. The game is played weekly with peers, whereby participants answer one alcohol-related and one nonalcohol-related question about their behavior. After 4 days, they receive normative feedback (ie, how their responses compared with their peers) and reflective evaluations from other students (ie, how they were judged by their peers). App use: questions and feedback delivered once each per week over 6 weeks. Adjunct components: None. App intervention 2: PNF—Same as app intervention above, with only normative feedback to alcohol questions (reflective evaluations were for nonalcohol-related questions).	Same as app intervention 1 condition, however, with no normative or reflective feedback on alcohol questions (normative feedback and reflective evaluations were given for nonalcohol-related questions in this condition). Comparison includes app? Yes	Duration: 6 weeks; assessments: baseline, 2 months; retention: 80% (PNF^+^); 84% (PNF)	Whole sample: age not provided; 55% female
Gajecki et al, 2014 [38]; “PartyPlanner”	University students reporting AUDIT>8 (men) or AUDIT>6 (women), risky drinkers; alcohol	PP^g^ app consists of (a) real-time feedback on eBACs^h^; (b) simulating a drinking event by entering predicted eBAC levels before an event; (c) the user records their alcohol consumption then compares the simulation with real-life event; (d) tracks how drinking compares with safe drinking. App use: no notifications, instruction to use before drinking events. Adjunct components: none. PK^i^ self-monitoring of alcohol; real-time eBAC feedback and alcohol-reduction strategies.	Assessment only comparison. Comparison includes app? No	Duration: 6 weeks; assessments: baseline, 7 weeks; retention: 61% (PP) and 74% (PK)	Whole sample: mean 24.72 (SD 4.81) years; 51.7% female
*Gajecki et al, 2017 [39]; “TeleCoach”*	University students reporting excessive alcohol consumption (>9 drinks per week for women; >14 for men); alcohol	“TeleCoach” (a) reporting of alcohol consumption for a week; (b) brief feedback and psycho-education; (c) a relapse prevention skills training, and guided relaxation and mindful “urge-surfing.” App use: no notifications, instructed to use at will. Adjunct components: eBAC app providing real-time feedback for 6 weeks before the intervention, with access during intervention.	Assessment only. Comparison includes app? No. However, as in the intervention condition, participants had access to another eBAC app for 6 weeks before, with access during intervention.	Duration: 12 weeks; assessments: baseline, 12 weeks; retention: 76%	Whole sample: mean 25.41 (SD 6.45) years; 69.1% female
Gonzalez and Dulin, 2015 [40]; “LBMI-A”	Adults who met the Diagnostic and Statistical Manual of Mental Disorders-5 alcohol dependence criteria; alcohol	“LBMI-A”: (a) assessment and feedback, (b) high-risk locations for drinking, (c) using supportive people for change, (d) managing cravings, (e) problem-solving skills, (f) communication or drink refusal skills, and (g) pleasurable nondrinking activities. App use: feedback reports delivered weekly, daily interviews to report alcohol consumption. Adjunct components: none	“Drinker’s Check-Up plus Bibliotherapy”, a web-based intervention that includes assessment of drinking. objective and normative feedback, decisional balance exercise, goal selection, development of change plan, and links to other web-based resources. Comparison includes app? No.	Duration: 6 weeks; assessments: baseline, 6 weeks; retention: 60%	Intervention: mean 33.57 (SD 6.54) years; 46.4% female; comparison: mean 34.30 (SD 6.22) years; 35.0% female
*Gustafson et al, 2014 [17]; “A-CHESS”*	Individuals who met the DSM-IV^j^ alcohol dependence criteria; alcohol	A-CHESS consists of (a) access to counselors, (b) a panic button related to relapse, (c) meditation, (d) recovery stories, (e) meeting locations, (f) recovery information, and podcasts App use: no notifications, instructed to use at will. Adjunct components: residential treatment.	TAU^k^ (support offered through the residential service); comparison includes app: No	Duration: 8 months; assessments: 4 months (during intervention, no baseline); 8 months; 12 months; retention: 78%	Intervention: mean 38.3 (SD 9.5) years; 39.4% female; comparison: mean 38.4 (SD 11.2) years; 39.1% female
Hasin et al, 2014 [41]; “HealthCall”	Adults who were HIV-positive and alcohol dependent; alcohol	“HealthCall” (a) alcohol self-monitoring; (b) consumption feedback compared with drinking goal, and feedback on drinks per drinking day and reasons for drinking. App use: one prompt per day re self-monitoring. Adjunct components: counselors administered a 25-min motivational interviewing session.	Same as intervention, but app replaced by HealthCall-IVR (a daily phone call using interactive voice response). Comparison includes app? No	Duration: 60 days; assessments: baseline, 60 days; retention: 90%	Intervention: mean 45.5 (SD 11.5) years; 28.2% female; comparison: mean 46 (SD 7.2) years; 18.6% female
Hertzberg et al, 2013 [42]; “mCM”	Adults with posttraumatic stress disorder who were regular smokers; tobacco	“mCM” (a) using a CO^l^ device to check abstinence; (b) using camera to record CO reading; (c) financial reward for each uploaded video showing “abstinent” CO, with progressive reinforcement schedule. App use: twice daily notifications for 4 weeks. Adjunct components: (a) two smoking cessation counseling sessions; (b) nicotine replacement therapy, low-nicotine cigarettes, and Bupropion; (c) 6 calls to assist withmotivation; (d) an additional 2 weeks of mCM app use, but without financial compensation.	Same app as intervention, but using noncontingent compensation, based on submitting videos of CO monitoring process, regardless of positive or negative CO reading. Comparison includes app? Yes	Duration: 4 weeks; assessments: 4 weeks, 3 months; retention: not reported	Intervention: mean 42.5 (SD 4.5) years; 36.4% female; comparison: mean 53.3 (SD 11.6) years; 27.3% female
Hides et al, 2018 [43]; “Ray’s Night Out”	People aged 16-25 years who drank alcohol at least monthly; alcohol	“Ray’s Night Out” (a) information on harm minimization strategies, (b) motivation to set a drinking goal, and (c) psychoeducation on consequences of intoxication. App use: no notifications used. Adjunct components: none.	Comparison procedure: waitlist. comparison includes app: No.	Duration: 1 month; assessments: baseline, 1 month; retention: 96%	Intervention: mean 20.4 (SD 2.2) years; 79.2% female; comparison: mean 20.5 (SD 2.5) years; 76.2% female
Kerst and Waters, 2014 [44]; “AR Training”	Adults who smoked 10 or more cigarettes per day for the past 2 years; tobacco	“AR Training” consists of attentional retraining (cognitive bias modification). Each training involves 160 trials. Trial begins with fixation cross on screen, followed by picture pair (smoking and neutral image), then dot probe. Required to indicate dot probe location quickly. App use: Four daily notifications (one assessment and three training) for 7 days. Adjunct components: none.	Same as app intervention, except dot probe equally likely to replace smoking and neutral images (no attentional bias modification). Comparison includes app? Yes.	Duration: 7 days; assessments: baseline, day 8; retention: 94%	Intervention: mean 41.8 (SD 10.2) years; 50% female; comparison: mean 43.6 (SD 14.0) years; 47% female
Krishnan et al, 2018 [45]; “Coach2Quit”	Daily smokers aged 18-years and above.	“Coach2Quit” uses real-time data from a carbon monoxide exhaler to provide users with tailored messages based on their CO result which is also graphically displayed. App use: twice daily notifications following CO breath test. Adjunct components: brief advice; c	Comparison procedure: brief advice only. Comparison includes app? No.	Duration: 30 days; assessments: baseline, day 14, day 30; retention: 87%	Intervention: Median 53 years; 59% female; comparison: Median 51 years; 58% female
Liang et al, 2018 [46]; “S-Health”	Adults from methadone maintenance clinics with heroin or other substances use in the past 30 days; drugs and alcohol	“S-Health” consists of daily surveys designed to serve as both assessment and intervention. Users respond to questions about (a) cravings; (b) affect; (c) trigger thoughts, places, and situations; (d) responses to triggers; and (e) social context. App use: daily notifications to complete surveys. Adjunct components: daily educational text message.	Daily educational text message (information about HIV prevention and other educational materials). Comparison includes app? No.	Duration: 4 weeks; assessments: 1 week, 2 weeks, 3 weeks, 4 weeks (no baseline); retention: 98%	Intervention: mean 41.7 (SD 8.7) years; 64% female; comparison: mean 41.3 (SD 6.8) years; 83% female
Ruscio et al, 2016 [47]; “Brief-MP”	Adults who smoked 10 or more cigarettes per day for the past 2 years; tobacco	“Brief-MP” consists of five audio-guided mindfulness sessions on (a) “urge-surfing” the craving, (b) mindfulness of the breath, body, thoughts, and emotions. Five daily assessments probed craving, mindfulness, and affect. App use: asked to meditate once per day. Four random daily assessment notifications and one following meditation session. Adjunct components: none.	Same as intervention, except sham-meditation recordings (eg, nonjudgmental awareness replaced with self-evaluation). Comparison includes app? Yes.	Duration: 2 weeks; assessments: baseline, 2 weeks; retention: 75%	Intervention: mean 45.34 (SD 11.84) years; 50% female; control: mean 44.16 (SD 13.64) years; 55% female
*Witkiewitz et al, 2014 [48]; “BASICS-Mobile”*	College students who engaged in at least one episode of heavy drinking during the past 2 weeks; alcohol and tobacco	“BASICS-Mobile” (a) daily monitoring, (b) normative feedback, (c) general or health information about drinking and smoking, (d) protective behavioral strategies, (e) alternative activities, (f) urge-surfing, and (g) decisional balance exercise. App use: 3 alerts per day for 14 days. Adjunct components: None. Comparison app: daily self-monitoring of alcohol consumption	Completed only initial screening, baseline assessment, and 1-month follow-up. Comparison includes app: No	Duration: 14 days; assessments: baseline, 1 month; retention: 94%	Whole sample: mean 20.5 (SD 1.7) years; 28% female

^a^Assessment time points reported here do not include assessments *during* the intervention.

^b^Retention is indicated only for the intervention group(s), defined as percentage completion of final (post intervention or follow-up) assessments.

^c^Studies in italics reported significant outcomes for intervention app at post intervention and/or follow-up compared with control. All studies delivered apps via smartphones, except for the study by Kerst and Waters [44], which used personal digital assistants (PDAs).

^d^AUDIT: Alcohol Use Disorders Identification Test.

^e^Factorial design; participants used either the enhanced or the minimal version of each component.

^f^The *Drink Less* app used a factorial randomized controlled trial design, whereby participants were randomized to 1 of the 32 groups, each receiving a different combination of intervention and comparison modules.

^g^PP: PartyPlanner.

^h^eBAC: estimated blood alcohol concentration.

^i^PK: Promillekoll.

^j^DSM-IV: Diagnostic and Statistical Manual of Mental Disorders, Fourth Edition.

^k^TAU: treatment as usual.

^l^CO: carbon monoxide.

**Table 2:** Summary of effects.

Study [reference]; target substance and substance use outcome measures		Intervention			Control				Between groups statistic and significance		Effect size (*d*)^a,b^		Quality assessment^c^	
		N	Pre, mean (SD) or n (%)	Post, mean (SD) or n (%)		n	Pre, mean (SD) or n (%)	Post, mean (SD) or n (%)						
***Aharonovich et al, 2017 [30]; drugs and alcohol^d^***														
	Number days primary drug use over 30 days	21	12.8 (4.4)	5.0 (4.7)		21	15.3 (7.3)	8.5 (6.1)		IRR^e^=.59 (.35-.99), *P*=.04		.17		Good
	Number drinking days over previous 30 days	21	14.2 (7.3)	7.0 (7.6)		21	13.7 (6.3)	8.1 (5.7)		IRR=.67 (.41-1.07), *P*=.09		.23		Good
	Standard drinks (14 g)^f^ per day over previous 30 days	21	3.2 (2.4)	.9 (1.0)		21	2.7 (2.0)	1.3 (1.1)		IRR=.63 (.36-1.11), *P*=.11		.41		Good
**Baskerville et al, 2018 [31]; tobacco**														
	Continuous abstinence at 6 months	354	0	49 (13.8)		371	0	57 (15.4)		OR^g^=.89 (.59-1.34), *P*=.56		.06		Good
	7-day point prevalence abstinence at 6 months	342	0	114 (33.3)		366	0	143 (39.1)		OR=.78 (.57-1.06), *P*=.11		.14		Good
	30-day point prevalence abstinence at 6 months	344	0	84 (24.4)		365	0	107 (29.3)		OR=.78 (.56-1.09), *P*=.14		.14		Good
**Boendermaker et al, 2015 [32]; alcohol^h^**														
	Standard drinks (10 g) over 14 days	25	25.1 (21.4)	20.0 (17.1)		24	25.4 (19.1)	18.4 (14.6)		*F*_1,44_=0.03, *P*>.05		–.05		Poor
**Bricker et al, 2014 [33]; tobacco**														
	Percent of sample abstinent over 30 days	80	(0) 0%	(10) 13%		84	0%	(7) 8%		OR=2.7 (.8-10.3), *P*=.12		.55		Fair
***Crane et al, 2018 [35]; tobacco***														
	Self-reported abstinence at 12-weeks	ITT^i^: 14,228; PP^j^: 1213	(0) 0%; (0) 0%	(234) 1.6%; (234) 19.3%		ITT: 13,884; PP: 901	(0) 0%; (0) 0%	(124) 0.9%; (124) 13.8%		OR=1.86 (1.49-2.31), *P*<.001; OR=1.50 (1.18-1.90), *P*<.001		.34; .22		Good
**Crane et al, 2018 [34]; alcohol**														
	Average number of alcohol units (8 g) per week	NF^k^:336; CB^l^: 336; MF^m^: 336; AP^n^: 336; IC^o^: 336; NFxCB^p^: 168	39.1 (25.97); 40.3 (28.23); 39.9 (27.09); 39.0 (26.46); 39.0 (26.62); 38.9 (26.92)	24.5 (22.45); 27.2 (25.96); 26.3 (23.41); 23.8 (24.23); 27.1 (26.47); 23.2 (20.68)		336; 336; 336; 336; 336; 168	40.7 (28.66); 39.6 (26.45); 39.9 (27.63); 40.9 (28.20); 40.8 (28.05); 39.9 (27.85)	26.6 (26.60); 23.9 (22.79); 24.5 (25.56); 27.0 (24.53); 23.7 (21.82); 21.5 (21.09)		*F*_1,178_=.30, *P*=.59; *F*_1,178_=.39, *P*=.53; *F*_1,178_=.78, *P*=.38; *F*_1,178_=.13, *P*=.71; *F*_1,178_=2.16, *P*=.14; *F*_4,43_=4.68, *P*=.03		.08; .09; .13; .05; .22; .67		Good
**Davies et al, 2017 [36]; alcohol**														
	AUDIT-C^q^ (alcohol consumption score)	104	6.6 (2.62)	6.0 (2.33)		OTM^r^: 99; IC^s^: 97; WL^t^: 102	6.2 (2.54); 6.8 (2.49); 6.6 (2.46)	5.7 (2.47); 6.1 (2.38); 5.9 (2.42)		All between-group comparisons *P*=ns		−.01; .03; .03		Good
***Earle et al, 2018 [37]; alcohol***														
	Maximum drink (undefined) number on single night over current semester	PNF+^u^: 72; PNF^v^: 79	4.23 (4.14); 3.87 (4.07)	2.97 (3.25); 3.53 (3.38)		71	3.80 (4.09)	3.82 (4.28)		See footnote^$^		.31; .09		Good
	Drink (undefined) number over previous weekend	PNF+: 72; PNF: 79	3.08 (4.10); 2.65 (3.74)	1.94 (2.67); 2.26 (3.28)		71	3.32 (4.81)	3.21 (4.70)		See footnote^$^		*.*23; .07		Good
	Composite score (maximum single occasion and drink number on previous weekend)	PNF+: 72; PNF: 79	3.08 (4.10); 2.65 (3.74)	1.94 (2.67); 2.26 (3.28)		71	3.32 (4.81)	3.21 (4.70)		Mean=−0.14, SE=0.07, *P*<.01^w^; mean=0.00, SE=0.07, *P*=ns^w^		.46; .24		Good
**Kerst and Waters, 2014 [44]; tobacco**														
	Percent of sample any smoking on test day	30	(29) 97%	(27) 90%		30	100%	(27) 90%		B=.04 (−.35 to .43), *P*=.85		−.01		Fair
	Expired carbon monoxide (ppm)	30	15.9 (5.35)	15.5 (7.70)		30	15.6 (4.68)	14.2 (5.81)		B=1.07 (−4.13 to 1.99), *P*=.49.		−.20		Fair
	Salivary cotinine (ng/ml)	30	394 (181)	410 (211)		30	420 (253)	408 (268)		B=*−*23.4 (*−*105.4 to 58.6), *P*=.57		−.13		Fair
**Gajecki et al, 2014 [38]; alcohol**														
	Standard drinks (12 g) over 7 days	PP^x^: 153; PK^y^: 341	8.57 (6.12); 9.62 (6.26)	8.32 (6.45); 9.75 (7.05)		489	9.15 (6.18)	8.62 (6.28)		Time x group LMM^z^ *P*=.82; time x group LMM *P*=.41		−.05; −.11		Fair
	Number drinking days over 7 days	PP: 153; PK: 341	2.17 (1.12); 2.24 (1.20)	2.17 (1.23); 2.36 (1.23)		489	2.29 (1.19)	2.15 (1.19)		Time x group LMM *P*=.23; time x group LMM Z=3.39, *P*=.001		−.12; ^−^.22		Fair
***Gajecki et al, 2017 [39]; alcohol^aa^***														
	Standard drinks (12 g) over 7 days	71	16.58 (7.84)	12.87 (9.73)		124	17.16 (7.87)	14.52 (7.46)		Z=−1.07, *P*=.29		.15		Fair
	Number drinking days over 7 days	71	3.35 (1.20)	2.51 (1.15)		124	3.53 (1.39)	3.02 (1.45)		Z=−2.12, *P*=.03		.30		Fair
**Gonzalez and Dulin, 2015 [40]; alcohol^aa^**														
	Standard drinks (14 g) over 7 days	28	39.12 (20.37)	22.07 (22.08)		20	33.34 (21.58)	12.26 (13.19)		PE=−4.28, *P<*.05		See footnote^ab^		Poor
	Percent heavy drinking days^ac^	28	54.25 (28.93)	26.98 (29.47)		20	47.74 (29.71)	15.04 (24.03)		PE=−8.25, *P<*.05		See footnote^ab^		Poor
	Percent abstinent days	28	30.36 (22.48)	51.32 (32.25)		20	38.21 (29.85)	60.90 (39.22)		PE=10.29, *P<*.05		See footnote^ab^		Poor
***Gustafson et al, 2014 [17]; alcohol***														
	Number of risky drinking days over 30 days^a^^d^	132	1.50 (0.34)^a^^e^	Post: 1.54 (0.49); FU^af^: 1.13 (0.50)		139	3.01 (0.48)^a^^e^	Post: 2.65 (0.48); FU: 2.60 (0.49)		Post mean difference=1.11, *P*=.10; FU mean difference=1.47, *P*=.03		−.18; .24		Poor
	Percent of sample abstinent over 30 days	132	(100) 75.6%^a^^e^	Post: (103) 78.1%; FU: (104) 78.7%		139	(94) 67.7%^a^^e^	Post: (93) 66.9%; FU: (91) 65.5%		Post between group OR=1.70, *P*=.04; FU between group OR=1.94, *P*=.02		.29; .37		Poor
**Hasin et al, 2014 [41]; alcohol**														
	Standard drinks (14 g) per drinking day over 30 days	39	9.3 (6.9)	3.9 (2.1)		43	8.1 (3.9)	3.6 (1.6)		See footnote^ag^		.16		Poor
	Percent of abstinent days over 30 days	39	58.1 (27.4)	79.2 (22.5)		43	61.3 (24.2)	82.1 (17.8)		See footnote^ag^		.01		Poor
**Hertzberg et al, 2013 [42]; tobacco**														
	Percent of sample abstinent for previous 7 days (bio-verified) at end of 4-week treatment	11	(0) 0%	(9) 82%		11	(0) 0%	(5) 45%		*Χ*^2^_1_=1.6, *P*=.21		.55		Poor
	Percent self-report (not bio-verified) abstinence at 3-months post intervention	11	(0) 0%	(6) 55%		11	0%	(2) 18%		*Χ*^2^_1_=2.0, *P*=.15		.64		Poor
**Hides et al, 2018 [43]; alcohol**														
	Risky single occasion drinking frequency over 1 month^ah^	97	2.11 (0.91)	2.23 (1.17)		86	2.10 (1.08)	2.25 (1.15)		*F*_1,184_=0.28, *P*=.60		.03		Fair
	Typical standard drinks (10 g) over 1 month	97	2.79 (1.41)	2.56 (1.32)		86	2.64 (1.40)	2.24 (1.21)		*F*_1,188_=1.00, *P*=.32		-.12		Fair
**Krishnan et al, 2018 [45]; tobacco**														
	Percent self-reported and biochemically verified abstinence at 30 days	39	(0) 0%	(1) 3%		50	0%	(1) 2%		*P*>.05		.04		Poor
	Median number of cigarettes over 30 days (IQR)	39	10.0 (6.0-20.0)	5 (4.0-10.0)		50	10.0 (8.0-20.0)	6.0 (3.0-10.0)		*P*=.84		See footnote^aj^		Poor
	Median expired carbon monoxide in ppm (IQR)	39	23.0 (18.0-33.0)	19.5 (15.0-26.0)		50	22 (14.0-30.0)	18.5 (10.0-28.0)		*P*=.52		See footnote^aj^		Poor
**Liang et al, 2018 [46]; drugs**														
	Number of days with primary drug use over 7 days (pre=end of week 1, no baseline)	48	1.33 (2.48)	0.71 (1.87)		25	3.08 (3.37)	2.20 (3.06)		OR=.29 (.06-1.44), *P*=.13 (regressed over 4 time points during intervention)		.09		Poor
	Drugs detected in urine (pre=end of week 1, no baseline)	42	(23) 56%	(11) 26%		20	(14) 70%	(10) 50%		OR=.57 (.11-2.84), *P=*.49 (regressed over 4 time points during intervention)		.11		Poor
**Ruscio et al, 2016 [47]; tobacco**														
	Cigarettes per smoking day	20	N/A	N/A		17	N/A	N/A		*F*_1,436_=5.50, *P*=.01^a^^k^		N/A		Fair
	Expired carbon monoxide (ppm)	18	18.4 (9.8)	14.3 (8.2)		14	19.1 (6.7)	15.4 (5.0)		*F*_1,29_=0.01, *P*=.92		.05		Fair
	Salivary cotinine (ng/ml)	18	504.4 (300.3)	433.9 (257.1)		14	452.9 (221.9)	482.8 (250.0)		*F*_1,29_=0.04, *P*=.84		.37		Fair
***Witkiewitz et al, 2014 [48]; alcohol and tobacco***														
	Standard drinks (14 g) per drinking day	BM^al^: 30; DM^am^: 29	BM: 5.57 (2.81); DM: 5.58 (2.45)	BM: 4.83 (2.59); DM: 4.56 (2.65)		26	7.46 (3.46)	6.05 (2.88)		*WALDX*^2^_2_=0.1, *P*=.94 ^an^		.08		Poor
	Number of heavy drinking days over 7 days^ao^	BM: 30; DM: 29	BM: 2.31 (1.53); DM: 2.45 (1.44)	BM: 2.07 (1.70); DM: 1.76 (1.33)		26	2.86 (1.41)	2.31 (1.35)		*WALDX*^2^_2_=0.1, *P*=.91^an^		.07		Poor
	Cigarettes per smoking day	BM: 30; DM: 29	BM: 4.93 (3.43); DM: 4.78 (4.83)	BM: 3.28 (3.35); DM: 2.71 (2.86)		26	3.76 (2.15)	4.55 (4.07)		B=2.04, *P*=.002; B=1.59, *P*=.02		.55^ap^; .45^ap^		Poor
	Number of days with drinking and smoking over 7 days	BM: 30; DM: 29	BM: 2.81 (0.59); DM: 2.82 (0.64)	BM: 1.97 (1.09); DM: 2.07 (0.88)		26	2.66 (0.48)	1.76 (0.83)		*WALDX*^2^_2_=2.0, *P*=.38 ^an^		.31		Poor

^a^All effect sizes are Cohen d. Sign of effect size indicates agreement with hypothesized direction (positive implies app condition improved outcome to a greater degree than comparison conditions; ie, a reduction in consumption or an increase in rates of abstinence).

^b^Where effect sizes not reported as Cohen’s d, effect sizes were converted from reported effect sizes where possible or derived using pooled baseline SDs from intervention and control groups, as described by Morris [49].

^c^Good quality=all criteria in the Cochrane Risk of Bias tool were met, fair quality=one criterion not met or two criteria unclear and the assessment that this was unlikely to have biased the outcome and there was no important limitation that could invalidate the results, poor quality=one criterion not met or two criteria unclear and the assessment that this likely biased the outcome and there were important limitations that could invalidate the results OR two or more criteria listed as high or unclear risk of bias.

^d^Studies in italics reported significant outcomes for intervention app at post-intervention and/or follow-up timepoints compared with control. Sample sizes reflect the number of participants included in the final analyses.

^e^IRR: incidence rate ratio.

^f^Amount in grams of pure alcohol in one standard drink varies across countries and is indicated in brackets.

^g^OR: odds ratio.

^h^Some data provided directly from authors.

^i^ITT: intention to treat analysis (referred to in publication as “Missing Equals Smoking”).

^j^PP: per protocol analysis (referred to in publication as “Follow-up Only”).

^k^NF: personalized normative feedback.

^l^CB: cognitive bias retraining.

^m^MF: monitoring and feedback.

^n^AP: action planning.

^o^IC: identity change.

^p^Two-way interaction between personalized normative feedback and cognitive bias retraining.

^q^First three questions of the 10-item Alcohol Use Disorder Identification Test, probing alcohol consumption.

^r^OTM: “One Too Many” (intervention website).

^s^IC: imagery control (sham website).

^t^wl: waitlist control.

^u^Intervention using personalized normative feedback plus reflection

^v^Intervention using personalized normative feedback without reflection.

^w^Main analysis used variables derived by combining drinking measures and controlling for a range of other covariates.

^x^PP: PartyPlanner app.

^y^PK: Promillekoll app.

^z^LMM: linear mixed model.

^aa^Some data provided directly from authors.

^ab^Group×time interaction analysis during intervention (not pre-post and hence was not considered superior to control as per our definition).

^ac^Heavy drinking defined here as 4+ standard drinks for females and 5+ for males.

^ad^Risky drinking defined here as 3+ standard drinks (14 g of alcohol) for females and 4+ for males consumed within a 2-hour period.

^ae^No baseline data were collected in this study as participants were inpatients who had not consumed alcohol for some time; authors use 4-month data as reference for 8-month post intervention and 12-month follow-up analyses.

^af^FU: follow-up.

^ag^ “No between groups significance conducted.

^ah^Risky single occasion drinking in this study is defined as 5+ standard drinks (10 g of alcohol) during one occasion.

^aj^Median scores, cannot compute Cohen d effect size.

^ak^LMM group×day interaction based on daily smoking reports over 2 weeks (not pre-post).

^al^BM: BASICS-Mobile app.

^am^DM: Daily monitoring app.

^an^Omnibus chi-square test across all 3 conditions.

^ao^Heavy drinking defined here as 4+ standard drinks for females and 5+ for males.

^ap^Effect size controlling for range of predictors.

^$^ Between group significance testing not conducted

**Table 3:** Summary of usability.

Study [reference]; app name	Usability measures	Usability
Aharonovich et al, 2017 [30]; HealthCall	Engagement—proportion of days used out of total possible days, and satisfaction—7 items rated 1 (low) to 5 (high).	Engagement: 95% (range 68.7%-100%) of the possible 60 days. Satisfaction: mean 4.5 (SD 0.8).
Baskerville et al, 2018 [31]; Crush the Crave	Four satisfaction items (used frequently, easy to use, well laid out, and confidence in using) measured on a 5-point Likert scale (“strongly agree” to “strongly disagree”), overall satisfaction item using same scale as above, an overall helpfulness item on a 10-point Likert scale.	Mean (SD): Used frequently: 3.6 (1.2); Easy to use: 2.3 (1.1); Well laid out: 2.5 (1.1); Confidence in using: 2.8 (1.1); Overall satisfaction: 2.6 (1.3); Overall helpfulness; 4.3 (2.7).
Boendermaker et al, 2016 [32]; Alcohol/Avoid	User experience measured on 5-point Likert scale, 1 (strongly disagree) to 5 (strongly agree).	Mean (SD): Ease of use:15.77 (2.11); Player enjoyment:13.19 (2.98); Player involvement Mean (SD) 11.23 (2.13); Task compliance:6.07 (1.53).
Bricker et al, 2014 [33]; SmartQuit	Treatment satisfaction measured on 5-point scale, 1 (not at all) to 5 (very much), and utilization: self-reported number of times opened app.	85% said app organized; 53% said app useful for quitting; 59% were satisfied overall; app mean use 37 times over 8 weeks (no prompts).
Crane et al, 2018 [34]; Drink Less	Four usability measures rated on a 5-point scale, 1 (not at all) to 5 (extremely).	Mean (SD): Helpfulness: NF^a^: 3.05 (0.88); CB^b^: 3.02 (0.98); MF^c^: 3.18 (0.93); AP^d^: 3.04 (1.02); IC^e^:3.09 (0.97). Ease of use: NF: 3.45 (0.97); CB: 3.45 (0.97); MF: 3.59 (1.00); AP: 3.56 (1.07); IC: 3.57 (1.00). Recommend: NF: 2.99 (1.23); CB: 2.91 (1.23); MF: 3.25 (1.22); AP: 3.08 (1.23); IC: 3.15 (1.16) . Satisfaction: NF: 3.22 (0.95); CB: 3.20 (0.97); MF: 3.36 (1.00); AP: 3.26 (1.00); IC: 3.25 (0.95).
Dulin et al, 2014 [50]; LBMI-A	Helpfulness and ease of use of each tool rated on a 7-point scale, 1 (extremely unhelpful or extremely easy to use) to 7 (extremely helpful or extremely difficult to use).	Mean (SD): Ease of use: 5.6 (1.7), with the Drink Monitor Tool being the easiest to use, 6.6 (1.8) and the High-Risk Location Tool being the most difficult, 4.3 (1.6). Helpfulness: High-Risk Location Tool was least helpful, 3.8 (2.2) and the Daily Interview Tool was most helpful, 6.1 (1.1).
Gajecki et al, 2014 [38]; PartyPlanner	Self-reported app usage and questions on ease of use, suitability, and likelihood of recommending to a friend, on a 5-point scale.	Mean (SD): Self-reported usage (any): PP^f^, 41.4%; PK^g^, 74.1%. Ease of use: PP: 3.2 (1.1); PK: 4.0 (1.1). Suitability: PP: 3.6 (1.2); PK 3.4 (1.2). Would recommend: PP: 3.6 (1.2); PK 3.7 (1.3).
Hasin et al, 2014 [41]; HealthCall	Twelve satisfaction questions on a 3-point scale, assessing feelings of safety and privacy, effects on recall and knowledge of own drinking patterns, motivation and self-confidence to reduce consumption, and app’s ability to prompt drinking goals.	Intervention group (“agree”): perceived responses as safe (94.59%), concerned about privacy (37.84%), liked using app (91.89%), graphs increased interest in app (86.49%), and graphs increased perceived benefit of app (91.89%). Of the 30 daily suggestions for cutting down drinking, 13 were rated as “helpful”/“very helpful” by over half the patients.
Hides et al, 2018 [43]; Ray’s Night Out	Mobile Application Rating Scale (5-point rating scale, 23 items), assessing engagement, functionality, aesthetics, and information quality.	Mean (SD): The MARS^h^ indicated the app had a good level of overall app quality: 3.82 (0.51); Functionality: 3.98 (0.69); Aesthetics: 4.03 (0.62); Information: 4.0 (0.56). Participants reported that they were unlikely to pay for the app:1.25, (0.69) and gave it a 3 out of 5-star rating: 3.13 (0.76).
Liang et al, 2018 [46]; S-Health	Seven usability questions (5-point scale), assessing ease of use, recall feasibility, willingness to provide responses, etc.	Intervention group (“strongly agree” or “agree”): “The survey questions were easy to understand” (55.3%); “I was comfortable answering these questions” (68.1%); “I was able to remember the number of days or frequency using alcohol or drugs in the past week” (53.2%); “The smartphone screen was easy to use” (72.3%); “I prefer to answer these questions myself on a cellphone instead of having a person ask me” (46.8%).
McTavish et al, 2012 [51]; A-CHESS	Passive app use data: which service selected; duration of use for each service; which pages viewed; messages sent or received.	93.5% accessed the system during the first week after leaving treatment. The A-CHESS services used by the greatest percentage of participants included *discussions*, *my messages*, *my team*, and *weekly surveys*. The least used services were *instant library*, *frequently* *asked questions*, and *web links*.
Pocuca et al, 2016 [52]; Ray’s Night Out	Mobile Application Rating Scale -youth version (5-point rating scale, 23 items), assessing engagement, functionality, aesthetics, and information quality.	Mean (SD): Entertaining: 3.78 (SD 0.83); Interesting: 3.67 (0.71); Customizable: 3.00 (0.58); Interactive: 2.63 (0.74); Speed and accuracy of function: 4.78 (0.44); Ease of use: 4.44 (0.73); Flow and logic: 4.33 (0.50); Layout design: 4.33 (0.50); graphics quality 4.56 (0.73); quality and credible information 3.71 (0.58); would you recommend 2.78 (1.09); overall star rating 3.11 (0.60).

^a^NF: personalized normative feedback.

^b^CB: cognitive bias retraining.

^c^MF: monitoring and feedback.

^d^AP: action planning.

^e^IC: identity change.

^f^PP: PartyPlanner app.

^h^MARS: Mobile App Rating Scale.

^g^PK: Promillekoll app.

### Risk of Bias

**Figure 2:**
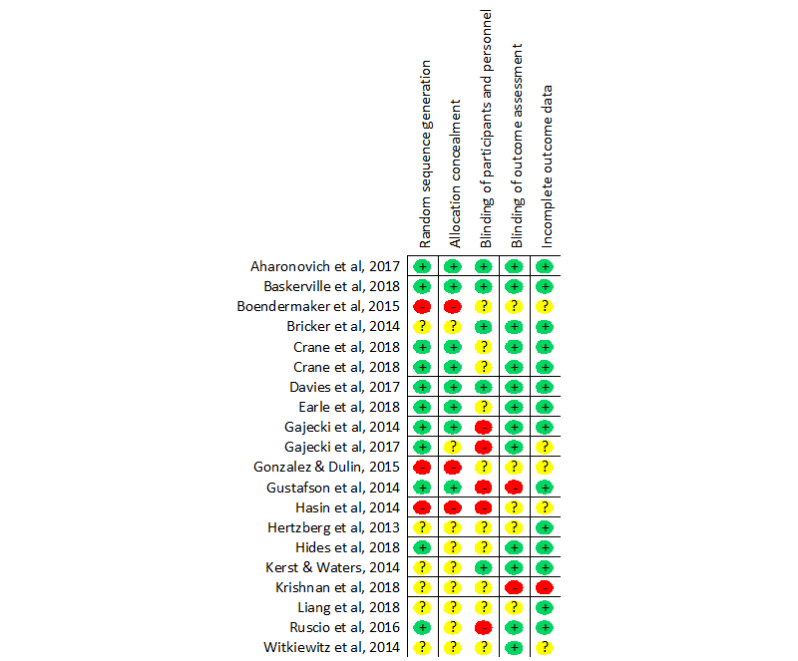
Risk of bias summary.

Risk of bias was assessed using the *Risk of Bias* tool developed by the Cochrane Collaboration [26]. This tool enabled the assessment of 5 key sources of bias: (1) allocation sequence, (2) allocation concealment, (3) blinding of participants and personnel, (4) blinding of outcome assessment, and (5) incomplete outcome data. The assessment of risk of bias for each study was conducted by 2 independent reviewers (JB and RB), and disagreement was resolved through discussion. Table 2 provides the quality ratings of the included studies; please refer to Figure 2 [17,30-48] for a summary of the sources of bias.

### Data Synthesis and Analyses

As stated, a decision was made a priori to not conduct a meta-analysis. Nonetheless, we report Cohen *d* effect sizes to enable meaningful comparisons and interpretations (Table 2). When effect sizes were not reported in the paper, we computed them using pooled baseline SDs from the intervention and control groups, as described by Morris [49].

## Results

### Design and Target Sample

A total of 20 studies met the inclusion criteria for the review. Eighteen studies were randomized controlled trials (RCTs) and two were matched controlled studies [39,41], as the control groups in each of these studies were from a related trial (conducted by the same research group), matched on main measures, and adopting the same eligibility criteria (ie, matched controlled studies). Specifically, in the study by Gajecki [39], participants in the comparison were from an assessment-only control group in a concurrent study that had the same eligibility criteria and were matched on alcohol consumption. In the study by Hasin [41], the matched controls were the control participants from a previous randomized trial adopting the same eligibility criteria.

The studies varied considerably in the sample size per group available for analysis, ranging from 22 to 14,228. The majority of studies were recruited from community samples with a wide age range, with the exception of 5 studies that included only university students. Five studies included a clinical or dependent sample [17,40-42,46], and gender tended to be evenly balanced, with the exception of the clinical studies, which had a greater proportion of males. Moreover, 10 apps targeted alcohol reduction[17,32,34,36,38-41,43,45], 7 targeted tobacco [31,33,35,42,44,47], 1 targeted alcohol and tobacco [48], 1 targeted drugs [46], and 1 targeted illicit drugs and alcohol [30].

### Risk of Bias

The methodological quality varied considerably. A total of 11 studies (11/20, 55%) reported adequate generation of random sequencing, 9 studies (9/20, 45%) reported adequately concealed group allocation, 5 studies (5/20, 25%) reported appropriate blinding of participants and personnel, 13 studies (13/20, 65%) reported appropriate blinding of outcome assessment, and 14 studies (14/20, 70%) adequately accounted for incomplete data. Overall, only 3 studies were classified as having a low risk of bias on all 5 measures of risk of bias. Moreover, 4 studies were classified as having a low risk of bias on 4 measures, 5 studies on 3 measures, 1 study on 2 measures, and 7 studies had low risk of bias on either none or only one measure. See Figure 2; more details regarding the source of bias and an in-text description of the risk of bias are provided in Multimedia Appendix 1.

### App Content, Complexity, and Supportive Components

Apps differed substantially in their intervention content. Most apps were stand alone, but 8 [17,30,31,39,41,42,45,46] of them had additional adjunct components such as supportive counseling; motivational interviewing; educational messages; links to resources; peer group supports such as Facebook groups; nicotine replacement therapy; audio-guided relaxation; and even a high-risk patient locator, which sends an alert to patients if they are approaching a high-risk drinking location (Table 1). In terms of app complexity, at least half of the apps consisted of multiple intervention elements [17,34,35,48]; others were simple and employed a single distinct intervention such as approach-bias training [44,47]. Apps varied substantially in the underlying theoretical approaches of their interventions (motivational interviewing, approach-bias modification, meditation, acceptance and commitment therapy, and relapse prevention). Finally, the majority of apps included self-monitoring of substance use as part of the intervention, and only 1 app (DrinkLess) directly tested the intervention components.

### Comparison Conditions

The comparison conditions were highly diverse. That is, Crane et al [34], Crane et al [35], Earle et al [37], Hertzberg et al [42], Kerst and Waters [44], and Ruscio et al [47] used a variant or minimal version of the intervention app, controlling for some key intervention component; Bricker et al [33] used an unrelated app; Boendermaker et al [32] and Hasin et al [41] used the same intervention but not delivered by an app; Gonzalez and Dulin [40], Liang et al [46], and Davies et al [36] used a nonapp different substance use intervention; Aharonovich et al [30] and Gustafson et al [17] used the adjunct or treatment as usual intervention as the comparison group (ie, minus the app); Baskerville et al [31] used information material and links to resources; and 5 studies used a waitlist, passive control or assessment only [36,38,39,43,48].

### Intervention Duration, Time to Follow-Up, Notifications, and Frequency of Contact

The intervention duration was generally short to medium length (1 to 8 weeks), with the exception of Gajecki et al (12 weeks) [39], Hides et al (26 weeks) [43], and Gustafson et al (35 weeks) [17]. *Smoke Free* and *Drink Less* did not specify an intervention length. Aside from 2 studies [17,42], no follow-up assessments were conducted following end-of-treatment measures. Most apps employed assessment and/or intervention notifications or alerts; however, 6 apps did not use notifications and instead requested that participants use the app at will or during specific events such as when drinking alcohol [17,32,33,38,39,43]. For the apps that did employ notifications, the most common schedule was once per day. Four apps used more frequent notifications 2 times per day [42], 3 times per day [48], and 4 times per day [44,47]. One app employed a single weekly email reminder [33].

### Effectiveness Outcomes

Cohen *d* effect sizes were extracted or calculated for each substance consumption outcome. For five studies where outcome data did not conform to the requirements for the calculation as described by Morris [49], effect sizes were computed by converting from the reported effect size to *Cohen d,* as indicated in Table 2. In three cases, insufficient data were provided (despite requests to authors) to calculate a pre-post effect size.

A minority of studies (6/20; Table 2) reported significant reductions in substance use compared with the comparison group at post treatment or follow-up.

Of the 6 apps that reported superior outcomes (compared to controls) at post treatment, 3 targeted alcohol, one of which was with clinical participants (A-CHESS), and the other two were focused  on university students (TeleCoach and CampusGANDR). One app was delivered to smokers (SmokeFree) and another targeted both alcohol and smoking in a university population (BASICS-Mobile), but in the latter study, only the smoking reductions (not alcohol) were superior to the comparison condition. Finally, one app (HealthCall) targeted illicit drugs and alcohol in an HIV population, but only a significant reduction in drug use (not alcohol) was reported in comparison to controls. It should be noted that most apps did report reductions in substance use; however, they were not necessarily superior to the control conditions post treatment. In addition, three apps were categorized as showing promise. Brief-MP (smoking app) and LBMI-A (alcohol app with a clinical group) reported intervention effects during treatment but not post treatment, and DrinkLess reported a significant reduction in alcohol consumption with a combination of two components (normative feedback and cognitive training).

Witkiewitz et al [48] reported on “BASICS-Mobile” that delivered monitoring, normative feedback, health information, alternative activities, and “urge-surfing” over 14 days focusing on alcohol and smoking reduction in university students. The app performed better for cigarettes smoked per day compared with a minimal control condition at post intervention (*d*=0.55), and no intervention effect was found for alcohol. Gustafson et al [17] showed that “A-CHESS,” which delivered psychoeducation, recovery stories, meeting locations, guided meditation, and access to phone counselors over 8-months, performed better than treatment as usual for 30-day alcohol abstinence (*d*=0.37) and number of risky drinking days (*d*=0.24). Aharonovich et al [30] showed that, alongside motivational interviewing, “HealthCall”—an app delivered over 8 weeks employing motivational self-monitoring, personalized feedback, and the option to call a phone counselor—produced significantly lower rates of primary drug use compared with a motivational interviewing only control group (*d*=0.17). Gajecki et al [39] reported that “TeleCoach”—an app that delivered alcohol monitoring, personalized feedback, alcohol guidelines and risk situations, drink refusal skills, and “urge-surfing” over 3 months—was associated with a reduction in drinking occasions (but not quantity) compared with a waitlist group (*d*=0.30). Earle et al [37] reported that the app “CampusGANDR”—a campus-based game that primarily centered on normative and injunctive feedback over 6 weeks—was associated with a reduction in drink number over a weekend compared with a control app that provided feedback about activity reports unrelated to drinking (*d*=0.23). Crane et al [35] reported that “Smoke Free”—an app consisting of goals, monitoring, daily messages that reported on accrued benefits (eg, financial savings and estimated health improvements), and behavior change strategies over a 30 day period—was associated with higher 3-month continuous smoking abstinence rates compared with a minimal version of the app (*d*=0.22 using per protocol analysis and *d*=0.34 using intention to treat analysis; see Multimedia Appendix 1 for details).

In addition, we categorized three further apps as showing promise. Brief-MP and LBMI-A were associated with significant reductions in the intervention arm, although this difference was no longer significant at post intervention (see Multimedia Appendix 1 for details) [40,47]. In addition, although *Drink Less* was associated with no overall difference between the intervention and control app, a significant interaction was found between the normative feedback and cognitive training components within the intervention group only, suggesting that these two components in combination resulted in a greater decrease in alcohol consumption compared with their minimal app (see Multimedia Appendix 1 for details). Given that this analysis was exploratory (although prespecified), we await further research before drawing any conclusions.

### Retention, Engagement, and Usability

Retention rates for the intervention group—usually defined as completion of final assessments (post or follow-up)—were generally good (70-80%) [17,39,47], high (80-90%) [32,33,37,42], or very high (over 90%) [30,41,43,44,46,48]. Lower retention rates were found in the studies by Gajecki et al (61%) [38], Gonzalez et al (60%) [40], and Crane et al (27%) [34]. See Table 3 for details.

Engagement—generally defined as responding to notifications or use in line with instructions (eg, in some trials, participants were instructed to use the app daily without providing notifications)—varied more, with some studies reporting low engagement (below 50%) [38,46], others reporting moderate engagement (50-80%) [47,51], and some reporting high user engagement (80% and over) [30,33,41,42,44].

Studies have used various methods to ascertain usability and user satisfaction, including reliable instruments such as the mobile application rating scale (MARS) [53], or a single item (Table 3). Satisfaction ratings ranged from moderate (50-80%) [33] to high (80% and over) [30,41]. For example, Hides et al [43] used the MARS and found that Ray’s Night Out had good objective app quality and high (80% and over) levels of functionality, aesthetics, and information. Hasin et al [41] reported high satisfaction, with 86% of patients stating that HealthCall-S reminded them of their drinking goal and over 80% stating that it increased confidence and motivation to reduce drinking. In the study by Bricker et al [33], 59% said they were satisfied overall.

In summary, of the 6 apps that were significantly more effective than their comparison conditions, all reported small to moderate effect sizes. Moreover, 3 of the 6 app studies were assessed as having a high risk of bias and 3 as having a low risk of bias; hence, no particular pattern emerged regarding outcomes and bias. When multiple substance consumption measures were reported, significant outcomes were mostly variable. Further details of each study are provided in Multimedia Appendix 1 and the tables.

## Discussion

The primary aim of this paper was to synthesize and report on an up-to-date systematic literature review focused on the effectiveness of substance use (alcohol, illicit drugs, or tobacco) interventions delivered via mobile apps. A total of 20 studies were included in the review, of which only 6 reported significantly greater reductions in substance use post intervention compared with comparison groups [17,30,35,37,39,48]. The average effect sizes were modest, although this is consistent with mobile apps in other fields, including mental health [15] and diet and exercise [54]. Two further trials [40,47] reported significant intervention effects during the treatment phase, with no significant group differences at post intervention. A third app reported a significant interaction for two intervention components within the app [34].

The 6 apps that performed significantly better than their comparison conditions varied substantially in intervention length, content, and complexity, and few commonalities across the majority of these emerged. In terms of app content, 3 of the 6 apps included normative feedback, and 1 app included personalized feedback (actual consumption compared with goals). Specifically, CampusGANDR rested heavily on personalized normative feedback and injunctive feedback (what peers think you should do); TeleCoach provided personalized normative feedback immediately following consumption reports; BASICS-mobile delivered normative feedback every day; and HealthCall included personalized feedback comparing actual consumption with personal goals. Interestingly, in the earlier study by Gajecki [38], the comparison condition, which included personalized normative feedback, performed better than the intervention, which did not deliver normative consumption feedback. This association between personalized feedback and normative feedback is consistent with previous face-to-face interventions demonstrating the effectiveness of these approaches [55] within mHealth approaches to substance reduction. Given the known importance of peers and normative attitudes in relation to substance use, including this component in future apps (particularly in young populations) may enhance efficacy.

In addition, the length of intervention may have played a role in influencing positive outcomes, with only 1 of the 6 highlighted interventions being under 6 weeks long (4, 6, 8, 12, and 35 weeks). In contrast, most of the studies that did not report intervention superiority ran for 4 or fewer weeks. Although only suggestive, it is possible that behavior change via an app may be more effective when the intervention component is greater than 4 weeks and participants engage for longer periods.

Retention rates were generally high across all studies, with the majority showing above 90% retention at postintervention or follow-up, except for the *Smoke Free* [35] and *Drink Less* [34] apps where retention at follow-up was less than 30%. The two latter studies differed from the rest as people enrolled in the study after having downloaded the app, as opposed to being recruited to a trial from the outset. This suggests that retention may be poor for apps used outside of research trials, and methods that enhance retention are of utmost importance if apps are to be effective as a public health approach. Moreover, 9 of the 20 studies reported usability data, with some variable results. Encouragingly, participants generally experience mHealth apps as easy and convenient to use. Considering that the poor usability of smartphone apps is common and can substantially compromise user engagement [56], these results are promising.

Four of the apps (LBMI-A, A-CHESS, Health Call, and Health S) were targeted at clinical samples who were primarily alcohol-dependent individuals, except for Health S (heroin addiction). Only 1 of the 4 reported superior outcomes (A-CHESS) [17]. This app intervention was for alcohol-dependent individuals who had already been in residential treatment, and hence, it functioned as a relapse prevention program. Furthermore, A-CHESS included adjunct components such as contact with counselors when required, indicating that the app alone was likely not responsible for the intervention effect. In addition, it is likely that this study has some risk of bias as it would have been clear to participants that they were in the intervention group given that the comparison group was treatment as usual with no additional support. It is not surprising given the complexity of alcohol and drug dependence that a mobile app may not result in significant positive outcomes for clinical samples, particularly given that most of the interventions except for A-CHESS were 6 weeks or less in duration. Although it is too early to draw any firm conclusions, it does appear that if mobile apps are helpful for those who are dependent on substances, it is likely to be most effective as posttreatment support rather than as the primary intervention. Interestingly, of the 7 apps that targeted tobacco use (1 targeted both alcohol and tobacco, BASICS), the only 2 reporting superior outcomes were *Smoke Free* and BASICS, which had no lower limit on smoking level, whereas the other trials included daily smokers, some of which were smoking 10 cigarettes a day, which would be considered in the mild to moderate dependence range. Once again, this tends to suggest that individuals with heavier substance use (if not clinical) are less likely to benefit from mobile apps. Given the small numbers, it is not possible to draw any conclusions regarding effectiveness in relation to the type of substance, although there is some suggestion that mobile apps are less effective with dependent individuals. We await further studies to confirm this conclusion.

Finally, it is important to note that the reductions in substance use produced by some of the app interventions were small in absolute terms. For example, compared with the comparison conditions, the app conditions with significant consumption outcomes produced mean reductions of one less day of drug use over 30 days [30], 0.8 of a day less drinking per week [39], 5% increase in the likelihood of being abstinent [17], and one less drink over a weekend [37]. Nonetheless, at a public health level, even small reductions at a population level can have a significant impact on the reduction of mortality and morbidity associated with problematic alcohol and other drug use and thus remain encouraging. Furthermore, although the majority of studies did not report “superior” outcomes (to their comparison conditions), in many cases, they reported significant decreases in alcohol or illicit drugs or tobacco. This will occur in study designs when comparison conditions are other apps or interventions delivered via other digital modalities (web-based and IVR) that we also know have a positive impact on substance reduction. In this respect, the mHealth field would benefit from greater consensus and clarity regarding the expectations of app efficacy and the role mobile apps should play in clinical treatment and public health approaches.

### Limitations

Numerous limitations were apparent in the included studies. For example, most studies were affected by design limitations and risk of bias and many were small sample pilot studies. That is, studies varied considerably in terms of sample size, with many small studies (eg, eight intervention conditions had samples of 30 or fewer); in contrast, two studies had samples greater than 1000.

One of the most significant limitations was that comparison conditions varied considerably, and in many cases were poorly balanced with the intervention condition. This variability in design reflects distinct kinds of research questions and precludes being able to draw any conclusions (tentative or otherwise) about the effectiveness of apps compared with other modes of delivery for problematic substances. Similarly, the 6 apps reporting superior outcomes compared with controls are confounded by substantially different kinds of comparison conditions—some likely to have very little therapeutic benefit (eg, waitlist control) and others comprised a similarly comprehensive “treatment” as the intervention condition (eg, web-based version of the same intervention). In addition, some studies included comparison conditions that were poorly balanced in terms of content and frequency of contact [17,30,46]. For example, in one study [17], participants in the treatment group had more counselor contact and completed a weekly assessment of alcohol intake, not delivered in the comparison condition, which may have produced an assessment effect. The Bricker et al study [33] included unspecified adjunct therapies (intervention group participants were encouraged to use other therapies alongside the trial, with no reporting of the details).

Finally, the risk of bias was generally high. At times, this was due to lack of detailed information, so it is possible that the true risk of bias could be lower across the studies. Overall, only 6 studies were classified as having either no or low risk of bias (Table 2; Multimedia Appendix 1). A further seven studies were assessed as potentially being biased, but a lack of information did not enable them to be classified as low risk. The remaining 8 studies were assessed as having a high risk of bias. Moreover, 3 of the 6 studies reporting superior outcomes were assessed as having no risk or low risk of bias, providing confidence that half of the significant findings were highly robust. Two of the superior trials had some risk of bias, of which one was due to unclear descriptions and one had high risk.

### Future Recommendations

This review highlights a number of key areas for future work in this fast-growing area. First, it is clear that we need sufficiently powered trials with longer follow-up periods and greater attention to reduce the potential risk of bias in these studies. An increasing focus on protocol papers, pre-registered trials, and adherence to Cochrane guidelines (and reporting thereof) will result in the ability to draw stronger conclusions in the next review.

Second, this review highlighted considerable variability in app content and complexity across a range of substances and inadequate descriptions of app content within publications. Furthermore, only half of the studies included descriptions of the user experience, which is critical to consider alongside the effectiveness data. If engagement and satisfaction from the user perspective is low, then the effectiveness outside of trial studies will be very low. The lack of usability data can be partly explained by word constraints and the reluctance of some journals to publish “user experience” papers. Thus, we recommend the field to engage with the Open Sciences Framework and similar platforms when providing details of app content, theories of change, and design.

In some cases, the development of app interventions was clearly described within the context of a theory of change for substance use reduction (eg, within the papers reporting on Smoke Free, Drink Less, and BASICS-mobile). However, in many cases, it was unclear what the proposed mechanism of change was and why it was chosen, and at times, it was difficult to ascertain the content of the intervention. In some cases, the rationale was to transfer “effective” face-to-face treatments to mobile apps (ie, BASICS-mobile), whereas other authors developed bespoke app interventions dependent on user input and the unique aspects of smartphone technology (ie, Ray’s Night Out). Ultimately, despite the substance use field having now produced 20 controlled evaluations of mobile apps, we remain unclear as to which “types” of interventions are likely to be most effective and the theory of change model underpinning them. Finally, in most cases, except for the DrinkLess app, there was little investigation of the effectiveness of the intervention components. The positive interaction between cognitive bias training and normative feedback found in the post-hoc analyses of the DrinkLess app is promising, given the ease by which both of these intervention components can be translated into a mobile app. Furthermore, the cognitive training component is a habit-forming activity and is well suited to an intervention that can be easily attended to on a smartphone at any time. Importantly, some behavior change interventions may be more aligned to digital delivery than others. For instance, we have recently proposed that a time-based goal setting technique rather than traditional count-based goals (to reduce smoking or alcohol or drug use) could be substantially enhanced by the unique capabilities of app functionality [57]. This might include daily reminders, timed alerts, automating reduction goals, supportive psychological strategies, and personalized delivery of interventions. As we continue to make further technological advancements in app delivery, well-aligned intervention content will be critical to the success of mHealth. A fine-grained analysis of the content of mobile apps in this field would be a helpful exercise in future publications.

Third, it was surprising to see a lack of iterative co-design processes being described in many of the publications (although Smoke Free, Drink Less, and Ray’s Night Out were some of the exceptions). Although potentially omitted in some cases due to manuscript length constraints, usability testing before evaluating the app in larger RCTs is critical. Such usability testing allows researchers to then modify the functionality based on user and clinician feedback, thereby avoiding inefficient or highly limited RCTs. Greater emphasis on co-design and usability testing will enhance our ability to improve retention. For example, we know that therapist guidance reduces attrition in digital interventions, but this can be costly. One possibility would be to trial automated guides or coaches to provide support and reduce attrition. Furthermore, the use of personalized reminders and strategies, machine-learning functionality, passive-sensor reporting, and context-based reminders have the potential to increase retention, in addition to other uses. However, none of the apps in this review incorporated these more complex and sophisticated technologies. We found this surprising, a pattern that could in part reflect funding constraints. Although evidence is lacking as to what level of collaboration is appropriate and at which point during the design process, it is likely that greater interdisciplinary and co-design collaboration, including users, researchers, clinicians, software developers, policy makers, marketing teams, and graphic designers, can produce more sophisticated products that will leverage these capabilities in the context of university research trials.

Finally, the considerable range of comparison conditions was a major limitation of this review, with the rationale for some of the chosen comparison conditions being somewhat perplexing. Each different type of comparison condition reflects a different research question and implies quite different purposes for an app focused on substance reduction. Researchers could consider whether their trial seeks to determine if the tested app will produce superior effects to an identical, similar, or different app intervention; computer intervention; face-to-face intervention; treatment as usual; or no intervention. At a public health level, we propose that if an app reduces substance use to the same degree as a more costly intervention, then this should be considered a positive trial outcome. This was not a discussion engaged in most of these papers and does point to a broader policy-based discussion as to what constitutes “app effectiveness” to ensure transparent communication with the public. Such considerations are important, given the potential broad reach that apps have in remote and financially disadvantaged communities or in addressing numerous other barriers to help seeking. The current saturation of smartphones in our society makes them a powerful mHealth tool, but further work is needed to understand how best to harness their capabilities, engage the user, and generate positive intervention outcomes. With hopeful anticipation, we look forward to what the next 5 years in mHealth research and development brings.

### Conclusions

It has been approximately 10 years since substance use interventions delivered via smartphone apps have become available, and the majority of controlled evaluations have been published in the last 5 years. As we are likely to see an acceleration in the development of smartphone app substance use interventions over the coming years, it is timely to take stock of the field and identify strengths, limitations, and future directions. This state-of-the-art review highlights the diversity in app design, with a range of options being explored for both community and clinical populations. The review also highlights substantial variability in study design, intervention types, comparison conditions, measures, follow-up period, length of intervention, and reporting details, making it almost impossible to infer factors or themes associated with the effectiveness of substance use apps specifically. We see this review as a *taking stock* moment; we are clearly not at the point where any firm conclusions can be drawn. Importantly, guidance from the details and outcomes of this review will hopefully strengthen the mHealth field in its future endeavors to assist individuals in the community to reduce their problematic consumption of alcohol, tobacco, and/or illicit drugs. Ultimately, we hope that mHealth can provide affordable, accessible, and effective behavior change interventions in this field.

Co-design is critically important in all intervention development; however, many studies do not incorporate the user until after important design and intervention decisions are made. To answer whether an app intervention is equivalent or superior in efficacy to other formats, the app should be tested against the same intervention delivered within a nonapp comparison condition. Ultimately, comparison conditions should be selected based on the fundamental research question. In addition to app-specific functionality that can be leveraged to produce innovative interventions, apps that demonstrate at least outcome equivalence compared with face-to-face treatment or treatment as usual would offer numerous advantages, including low cost, accessibility, reduced barriers to help seeking, and potentially higher engagement. Relatedly, efficacious app interventions that are able to recruit individuals otherwise unwilling to seek help would also offer substantial advantages in addressing the treatment gap. Indeed, app interventions have generated considerable interest in public health research, with some promising signs emerging from mental health apps [15], although see study by Weisel et al [16]. However, a similar story cannot yet be told for apps focused on helping people reduce problematic alcohol, tobacco, and/or illicit drug use. Although the field is still in its infancy, this review cautiously suggests that app interventions for problematic substance use are yet to clearly demonstrate their utility. In particular, and not surprisingly, this seems to be the case for clinical or heavier users of substances. A more positive state of the literature in the next review is likely to be enabled by greater collaboration between multidisciplinary teams, iterative learning from each other’s products, selecting evidence-based and mobile app–aligned content, greater expert and consumer input, attention to reducing risk of bias, comprehensive usability testing, more personalized interventions, and methods that leverage greater user engagement and retention.

## Appendix

Multimedia Appendix 1 Supplementary materials.

## Abbreviations

CONSORT-EHEALTH: Consolidated Standards of Reporting Trials-Electronic Health Checklist
IVR: interactive voice response
MARS: mobile application rating scale
mHealth: mobile health
RCT: randomized controlled trial
