# Supplementary material for: Mobile Apps to Reduce Tobacco, Alcohol, and Illicit Drug Use: Systematic Review of the First Decade
Source: J Med Internet Res. 2020 Nov 24;22(11):e17156. doi: 10.2196/17156 (PMC7723745; doi:10.2196/17156)
Supplement: Multimedia Appendix 1 [file jmir_v22i11e17156_app1.docx]

# Multimedia Appendix 1

**Search Strategy**

Key term 1: Substance

*“Alcohol*”* OR “*drug**” OR “*heroin**” OR “diamorphine” OR “*opiate*” OR “*opioid*” OR “*cocaine*” OR “*marijuana*” OR “*cannabis*” OR “*weed*” OR “*speed*” OR “*amphetamine**” OR “*methamphetamine**” OR “*sedative**” OR “*hallucinogen**” OR “*ecstasy*” OR “*mdma*” OR “*inhalant**” OR “*ketamine*” OR “*polydrug*” OR “*drink**” OR “*binge*” OR “*beer**” OR “*prescription*” OR “*booze*” OR “*liquor*” OR “*narcotic**” OR “*pill**” OR “*stimulant**” OR “*depressant**” OR “*benzodiazepine**” OR “*morphine*” OR “*diazepam*” OR “*tobacco*” OR “*cigarette**” OR “*substance**” OR smok*

EMBASE

*Alcohol** OR *drug** OR *heroin** OR diamorphine *OR opiate** OR *opioid** OR *cocaine* OR *marijuana* OR *cannabis* OR *weed* OR *speed* OR *amphetamine** OR *methamphetamine** OR *sedative**OR *hallucinogen** OR *ecstasy* OR *mdma* OR *inhalant** OR *ketamine* OR *polydrug* OR *drink** OR *binge* OR *beer** OR *prescription* OR *booze* OR *liquor* OR *narcotic** OR *pill** OR *stimulant** OR *depressant** OR *benzodiazepine** OR *morphine* OR *diazepam* OR *tobacco* OR *cigarette** OR *substance** OR smok*

Key term 2: Smartphone Delivered

AND "*ecological momentary**" OR "*just in time adaptive* " OR *JITAI* OR "*smartphone**" OR “*smartphone app**” OR "*mobile phone**” OR "*mobile phone app**" OR "*cell phone*" OR “*cell phone app**” *OR “tablet* device*” OR “ipad” OR “mobile device”*

EMBASE

AND ‘*ecological momentary**’ OR ‘*just in time adaptive*’ OR *JITAI* OR *smartphone** OR ‘*smartphone app**’ OR ‘*mobile phone**’ OR ‘*mobile phone app**’ OR ‘*cell phone*’ OR ‘*cell phone app**’ *OR ‘tablet* device’ OR ipad* OR ‘mobile device’*

Key term 3: Intervention

AND *intervention** OR *prevention** OR *therap** OR *treatment** OR RCT OR “*randomized control trial*” OR “*randomized controlled trial*” OR “*randomised control trial*” OR “*randomised controlled trial*” OR *program** OR *strateg** OR *technique** OR “supported recovery”

EMBASE

AND *intervention** OR *prevention** OR *therap** OR *treatment** OR RCT OR ‘*randomized control trial*’ OR ‘*randomized controlled trial*’ OR ‘*randomised control trial*’ OR ‘*randomised controlled trial*’ OR *program** OR *strateg** OR *technique** OR “supported recovery”

*Databases*

Medline, PsycInfo, Embase (via the OVID platform) and ERIC (via EBSCO).

**Detailed description of the “superior” mobile applications**

Below is a detailed description of the apps and the evaluation outcomes of the six superior interventions (to control) at post and/or follow up. This description also describes in more details the risk of bias information. The accompanying figure of risk of bias provides the overall summary.

In the Witkiewitz et al. study [1], a mobile feedback intervention targeting heavy episodic drinking and smoking, ‘BASICS-Mobile’ was evaluated using a sample of non-treatment-seeking college students. The intervention was delivered for 14 days and consisted of monitoring using three randomly-spaced assessments per day in addition to assessments that were to be completed before and after a drinking occasion; and post-assessment modules which delivered, for example, normative feedback, general or health information about drinking and smoking, and alternative activities to drinking and smoking. While controlling for all other predictors and compared with the minimal assessment condition, the number of cigarettes per smoking day was lower in both the BASICS-Mobile intervention (B = 2.04, *P* = .002, *d* = 0.55) and in the Daily Monitoring app condition (B = 1.59, *p* = .02, *d* = 0.45). However, it should be noted that a quality assessment of this trial, using the Cochrane Risk of Bias tool, was unable to determine the risk of bias as many of the sources of potential bias (for example, selection bias and reporting bias) were not reported in the paper and therefore, it was unclear whether the authors took the necessary steps to address these issues.

The Gustafson et al. study [2] reports on one of the first alcohol apps to be evaluated in an RCT, ‘A-CHESS’. They provided the app, a phone, and a data plan to a group of patients leaving residential treatment for alcohol dependence; the comparison group received treatment as usual. A-CHESS delivers a range of modules, including recovery psychoeducation, recovery stories, meeting locations, guided meditation, and access to phone counsellors. The app was used on-demand with no notifications and no self-monitoring. These interventions were provided for a period of 8 months. Improvements were significantly greater in the app condition than in the comparison for 30-day alcohol abstinence (*d* = .37) and number of risky drinking days (*d* = .24) at 4-month follow-up, and this effect was maintained 8 months later. However, it should be noted that a quality assessment determined this trial to be poor quality as it had a high risk of bias in one domain: the authors employed an unmasked randomised controlled trial design and furthermore it was unclear whether the researchers were blinded to participant group as they phoned participants to administer the outcome surveys. Given it is likely that participants would inadvertently report on their use of the phone it would become clear that they were intervention participants. Further, the intervention participants regularly reported on their alcohol consumption which as the authors point may have contributed to the effectiveness of the intervention.

HealthCall was developed by Aharonovich and colleagues [3] for HIV-positive patients reporting at least one binge drinking episode and/or drug use in the previous month. The intervention consisted of two in-person sessions of Motivational Interviewing, alongside the app. The app was delivered over 2-months with daily notifications, and employed a range of daily questions designed to increase behavioural self-awareness (substance use, risky behaviour, medicine adherence), while others reinforced goals, reasons for reducing substances, and enhancing self-efficacy. In addition, the app provided ongoing personalised consumption feedback, and allowed people to phone a counsellor if needed (uptake of this was not reported). Those in the app group reported significantly lower rates of primary drug use (*d* = .17) compared with the control group (who received two in-person sessions of Motivational Interviewing only) at the end of the two months, with no difference between the app intervention and control for alcohol consumption. Translating results to real world numbers, the app condition resulted in a difference (intervention vs control) of 3.5 fewer drug use days over a 30-day period when assessed at the end of the intervention. Using the Cochrane Risk of Bias tool we found no evidence of bias, such as selection bias, reporting bias, or outcome assessment bias. In this respect we are confident that the design and outcomes reported are robust.

TeleCoach was developed by Gajecki et al. [4] for a university student sample who self-reported excessive drinking. TeleCoach used daily alcohol monitoring followed immediately by personalised feedback and guidelines for hazardous drinking levels. In addition, the app provided a menu that included drink refusal skills, increasing awareness of situations that pose a personal risk for excessive consumption, and audio modules that included guided relaxation and ‘urge-surfing’. The intervention ran for 12 weeks, with no notifications sent. To note, participants in this study were drawn from those in a previous 18-week eBAC study who continued to drink excessively. They found that compared to a Waitlist group, alcohol frequency (but not quantity) was significantly reduced in the TeleCoach app group (*d* = .30). No follow-up data was reported and the intervention effect for those using TeleCoach translated into a reduction of 0.8 of a drinking day per week compared in the Waitlist condition. However, it should be noted that a quality assessment determined this trial to have one source of bias and another potential source. That is, participants were not blinded to the intervention they were in (i.e., it was a wait list design) and secondly it was unclear whether there was bias due to incomplete outcome data. It was not clear whether the differential attrition rate between the intervention and waitlist group could be a source of bias. Further information was required regarding reasons for attrition to make a clear judgement. At this stage it was evaluated as potentially some risk of bias thus making it difficult to be confident with the findings.

CampusGANDR, evaluated by Earle et al. [5], was offered to university students (no consumption eligibility criteria) as a campus-based game for 6 weeks. Each week, respondents would receive two notifications. For the first, they answered two questions about their past-week behaviour (one being about the number of drinks consumed, the other about their sexual activity), and were also asked to make personal judgements of hypothetical behaviours (including various levels of alcohol consumption). For the second notification a few days later, participants received two types of feedback: normative (e.g., group mean consumption) and injunctive (e.g., proportion of group with specific negative judgements about participant’s own behaviour). In the full app condition, participants received both normative and injunctive feedback about their alcohol consumption; in the comparison app, they received this feedback about their sexual behaviour only. At 6 weeks, a statistically significant reduction in number of drinks consumed over the previous weekend was reported in the full app condition compared to the comparison app (*d* = .23). No follow-up data was reported. The intervention effect translated to an average decrease of 1.1 drinks over a weekend in comparison to an app that provided feedback on unrelated behaviours. Using the Cochrane Risk of Bias tool, a quality assessment of this trial determined no evidence of bias indicating our confidence in the outcomes are strong.

‘Smoke Free’ was designed by Crane et al. [6] for adult smokers who wished to quit. The intervention lasted 30 days and consisted of setting goals, monitoring progress, and delivering daily messages that reported on the benefits achieved to date, such as financial savings and estimated health improvements. Despite very low follow-up rates, they found that the full version of the Smoke Free app resulted in higher self-reported 3-month continuous smoking abstinence rates compared to the reduced version (identical to the full version except for ‘daily missions’ which involved behaviour change techniques to avoid and resist cravings). Those in the intervention group were 1.5 times more likely to be abstinent 12 weeks after commencing the app intervention. A quality assessment of this trial determined low risk of bias despite the high attrition rate. The combination of the robust findings and low risk of bias provide confidence in the true findings of this study.

**Detailed description of three other apps that indicated some positive findings**

The three studies below show some promise but it should be noted that the LMBI-A [7] intervention for alcohol dependence and the Brief MP [8] app intervention for heavy smokers only reported significantly superior effects **during the intervention** which was no longer found post-intervention. The LMBI-A app was assessed reported as having a high risk of bias whilst the Brief MP app had two potential risks of bias but it was unclear. In contrast, the DrinkLess intervention had low risk of bias and though it did not report an overall intervention effect two intervention components in combination did report superior outcomes.

DrinkLess app was designed by Crane and colleagues [9]. It was the only study that adopted a factorial design which enabled the examined of interventions components as well as comparing outcomes against a minimal version of the app. The intervention components of ‘Drink Less – enhanced version’ consisted of: (a) goal-setting; (b) Personalized Normative Feedback; (c) Cognitive Bias Re-training; (d) Self-monitoring and Feedback of consumption and other harms; (e) Action Planning; (f) Identity Change. (Note: factorial design; participants used either the enhanced or the minimal version of each component). Individuals were sent daily notifications to report consumption for 4-weeks and they were provided with the use of intervention modules although with minimal explanation. Comparison procedure components: ‘Drink Less – minimal version’ consisted of: (a) goal-setting; (b) Alcohol consequences psychoeducation; (c) Sham Cognitive Bias Re-training; (d) Consumption Self-monitoring; (e) Information only about Action Planning; (f) Information only on role of identity in problematic habits, and advice to consider consequences. Both apps reported decreases in alcohol consumption with the enhanced version not reporting superior outcomes compared to the minimal version. Two components (personalised normative feedback and cognitive bias re-training) in interaction did report significant outcomes (*d* = .67). The quality of the study was high but given the number of interactions tested (despite them being pre-specified) caution must be taken when considering these results given the potential for Type I error.

In the Gonzalez and Dulin study [7], while the intervention group showed more rapid decreases in drinks per week, at *post-*intervention no significant differences were found between groups using raw scores (provided by authors). Also, while percent days abstinent increased significantly only in the intervention group (B = 13.30, *P* <.001), no significant group differences were found *post*-intervention. However, a quality assessment determined this trial to be poor quality as the participants were not randomly allocated into the study conditions and no allocation concealment occurred which allowed the investigators to foresee condition assignments. Finally, it was unclear whether the investigators addressed any other sources of bias in their methodology such as blinding and the management of missing data. Together the poor quality of the design and the lack of significant post-intervention outcomes we are less confident in the positive findings of this study.

Ruscio et al. [8] developed a mindfulness app to assist heavy smokers (more than 10 cigarettes a day for two years) to reduce their cigarette consumption. ‘Brief-MP’ consists of five 20-minute audio-guided mindfulness sessions focused on: (a) ‘urge-surfing’ the craving; (b) mindfulness of the breath; (c) mindfulness of the body; (d) mindfulness of thoughts; (e) mindfulness of emotions. People were instructed to complete one meditation per day at time of choosing for a two period. In addition, five daily assessments probed craving, state mindfulness, and positive/negative affect. Four identical daily assessment notifications randomly dispersed across the day, and one self-initiated assessment following meditation session. The comparison app was the same as the intervention, except meditation recordings were replaced with sham-meditation recordings (e.g., non-judgmental awareness replaced with self-evaluation). They used Linear Mixed Modelling to analyse number of cigarettes smoked per day, and showed a significant experimental condition by day interaction over the two-week trial, with a decline found only in the intervention group, *F*(1, 19) = 18.0, *P* = .0004. However, as number of cigarettes smoked per day was not reported at baseline or post-intervention, differences at post-intervention could not be assessed and authors did not respond to requests for further information. A quality assessment of this trial found it to be of fair quality. Although participants were randomised using a blocked procedure it was not clearly whether the personnel were concealed from knowing allocation. Further, even though participants were blinded to knowing the intervention they were in it was not made clear whether this was the case for the personnel. At this stage this intervention shows promise but it is possible that effect is only apparent during the intervention period.

Drink Less was an app downloaded by treatment seekers who then had the option of enrolling in the RCT [9]. This study was a between-subjects full factorial RCT that randomised risky drinkers to one of 32 experimental conditions, each receiving a different combination of five intervention components and five control components (see above as smoking intervention had same components). They found no main effect of group on reduced number of alcohol units per week. However, a two-way interaction between Personalised Normative Feedback and Cognitive Bias Re-training was found on average weekly alcohol quantity (*d* = .67). As the authors note, given the large number of interactions reported, this needs to be interpreted with caution. A quality assessment of this trial determined there to be low risk of bias.

# References

1. Witkiewitz K, Desai SA, Bowen S, Leigh BC, Kirouac M, Larimer ME. Development and evaluation of a mobile intervention for heavy drinking and smoking among college students. Psychology of Addictive Behaviors. 2014;28(3):639.

2. Gustafson DH, McTavish FM, Chih M-Y, Atwood AK, Johnson RA, Boyle MG, et al. A smartphone application to support recovery from alcoholism: a randomized clinical trial. JAMA psychiatry. 2014;71(5):566-72.

3. Aharonovich E, Stohl M, Cannizzaro D, Hasin D. HealthCall delivered via smartphone to reduce co-occurring drug and alcohol use in HIV-infected adults: A randomized pilot trial. Journal of substance abuse treatment. 2017;83:15-26.

4. Gajecki M, Andersson C, Rosendahl I, Sinadinovic K, Fredriksson M, Berman AH. Skills training via smartphone app for university students with excessive alcohol consumption: a randomized controlled trial. International journal of behavioral medicine. 2017;24(5):778-88.

5. Earle AM, LaBrie JW, Boyle SC, Smith D. In pursuit of a self-sustaining college alcohol intervention: Deploying gamified PNF in the real world. Addictive behaviors. 2018;80:71-81.

6. Crane D, Ubhi HK, Brown J, West R. Relative effectiveness of a full versus reduced version of the 'Smoke Free' mobile application for smoking cessation: an exploratory randomised controlled trial. F1000Res. 2018;7:1524. doi:10.12688/f1000research.16148.2

7. Gonzalez VM, Dulin PL. Comparison of a smartphone app for alcohol use disorders with an internet-based intervention plus bibliotherapy: A pilot study. Journal of consulting and clinical psychology. 2015;83(2):335-45. doi:10.1037/a0038620

8. Ruscio AC, Muench C, Brede E, Waters AJ. Effect of brief mindfulness practice on self-reported affect, craving, and smoking: a pilot randomized controlled trial using ecological momentary assessment. Nicotine & Tobacco Research. 2016;18(1):64-73.

9. Crane D, Garnett C, Michie S, West R, Brown J. A smartphone app to reduce excessive alcohol consumption: Identifying the effectiveness of intervention components in a factorial randomised control trial. Scientific Reports. 2018;8:4384. doi:10.1038/s41598-018-22420-8
